# Flurbiprofen Axetil Enhances Analgesic Effects of Sufentanil and Attenuates Postoperative Emergence Agitation and Systemic Proinflammation in Patients Undergoing Tangential Excision Surgery

**DOI:** 10.1155/2015/601083

**Published:** 2015-07-27

**Authors:** Wujun Geng, Wandong Hong, Junlu Wang, Qinxue Dai, Yunchang Mo, Kejian Shi, Jiehao Sun, Jinling Qin, Mei Li, Hongli Tang

**Affiliations:** ^1^Department of Anesthesiology, First Affiliated Hospital of Wenzhou Medical University, Wenzhou, Zhejiang 325000, China; ^2^Department of Gastroenterology, First Affiliated Hospital of Wenzhou Medical University, Wenzhou, Zhejiang 325000, China; ^3^Department of Anesthesia, Guangdong Provincial People's Hospital and Guangdong Academy of Medical Sciences, Guangzhou 510000, China

## Abstract

*Objective*. Our present study tested whether flurbiprofen axetil could reduce perioperative sufentanil consumption and provide postoperative analgesia with decrease in emergency agitation and systemic proinflammatory cytokines release.* Methods*. Ninety patients undergoing tangential excision surgery were randomly assigned to three groups: (1) preoperative dose of 100 mg flurbiprofen axetil and a postoperative dose of 2 *μ*g/kg sufentanil and 10 mL placebo by patient-controlled analgesia (PCA) pump, (2) preoperative dose of 100 mg flurbiprofen axetil and a postoperative dose of 2 *μ*g/kg sufentanil and 100 mg flurbiprofen axetil by PCA pump, and (3) 10 mL placebo and a postoperative dose of 2 *μ*g/kg sufentanil and 10 mL placebo by PCA pump.* Results*. Preoperative administration of flurbiprofen axetil decreased postoperative tramadol consumption and the visual analog scale at 4, 6, 12, and 24 h after surgery, which were further decreased by postoperative administration of flurbiprofen axetil. Furthermore, flurbiprofen axetil attenuated emergency agitation score and Ramsay score at 0, 5, and 10 min after extubation and reduced the TNF-*α* and interleukin- (IL-) 6 levels at 24 and 48 h after the operation.* Conclusion*. Flurbiprofen axetil enhances analgesic effects of sufentanil and attenuates emergence agitation and systemic proinflammation in patients undergoing tangential excision surgery.

## 1. Introduction

Burn patients usually suffered from tremendous pain [1–3], especially after tangential excision surgery. Good postoperative analgesia may help reduce pain related multiple problems, such as anxiety, emergency agitation, and lack of confidence in medical team [4]. Moreover, burn patients are predisposed to sepsis and multiple organ failure [5, 6]. These are major complications associated with burn trauma and the activation of a proinflammatory cascade after burn injury appears to be important in their development [7, 8]. Therefore, effective modulation of pain relief and the postoperative systemic inflammatory response should be beneficial to the patients who received tangential excision surgery.

Flurbiprofen axetil, which is incorporated in lipid microspheres, is a nonsteroidal anti-inflammatory drug (NSAID) with high affinity to the site of surgical incision and inflammatory tissues [9]. NSAIDs exert their analgesic effect not only through peripheral inhibition of prostaglandin synthesis but also through a variety of other peripheral and central mechanisms that augments the peripheral mechanism [10]. NSAIDs and opioid drugs are known to possess synergistic analgesic effects. It has been reported that pretreatment with flurbiprofen axetil enhances analgesic effect of fentanyl and reduces fentanyl concentration required for immobility under propofol anesthesia [11, 12]. Further studies show that preoperative intravenous administration of flurbiprofen axetil reduces postoperative pain in patients undergoing radical resection of esophageal carcinoma, postmastectomy, spinal fusion, and thyroid gland surgery [13–17] and relieves cancer related multiple breakthrough pain [18]. However, there are a few reports on whether preoperative flurbiprofen axetil can reduce perioperative opioid consumption and postoperative emergency agitation and pain after tangential excision surgery. In the present study, we hypothesized that preoperative flurbiprofen axetil can reduce perioperative sufentanil consumption and provide postoperative analgesia with decrease in emergency agitation and systemic proinflammatory cytokines release in patients undergoing tangential excision surgery.

## 2. Materials and Methods

### 2.1. Patients' Selection

This randomized, double-blind, and placebo-controlled clinical study (clinical trial registration number: ChiCTR-IPR-15005830) was approved by the Ethics Committee of the Wenzhou Medical University, and informed consent was obtained from the patients prior study enrollment. Ninety patients undergoing tangential excision surgery were involved in this study. The inclusion criteria were (1) American Society of Anesthesiologists (ASA) I-II and (2) age 25–55 years. The exclusion criteria were (1) patients with a history of allergic reaction to NSAIDs or opioid drugs, (2) patients with any contraindications for the use of NSAIDs, (3) patient with infections and inhalation injury, (4) patients with severe hepatic, renal, cardiovascular, or psychological disorders, and (5) patients with mental illness, neuromuscular diseases, language disorders, and immunodeficiency diseases.

### 2.2. Study Design

On the day before the surgery, all the patients were instructed about the study protocol and the use of visual analog scale (VAS) and the patient-controlled analgesia (PCA) pump. Patients who were unable to use the VAS and the PCA pump were excluded. The patients undergoing tangential excision surgery were randomly divided into three groups (*n* = 30 in each group). Group A patients received a preoperative dose of 100 mg [11] flurbiprofen axetil (Taide pharmaceutical Co., Beijing, China) by intravenous administration and a postoperative dose of 2 *μ*g/kg sufentanil (Renfu Pharmaceutical Co., Yichang, China) and 10 mL placebo (intralipid) and normal saline (total volume, 100 mL) by PCA pump. Group B patients received a preoperative dose of 100 mg flurbiprofen axetil by intravenous administration and a postoperative dose of 2 *μ*g/kg sufentanil and 100 mg flurbiprofen axetil and normal saline (total volume, 100 mL) by PCA pump. Group C patients received 10 mL placebo by intravenous administration and a postoperative dose of 2 *μ*g/kg sufentanil and 10 mL placebo and normal saline (total volume, 100 mL) by PCA pump. Preoperative flurbiprofen axetil/placebo was administrated 15 min before the induction of anesthesia.

### 2.3. Anesthesia and Analgesia Procedures

All patients involved in the study received intramuscular injections of diazepam 10 mg and atropine 0.5 mg at 30 min before anesthesia. On arrival at the operating room, standard monitors including pulse oximetry, electrocardiogram, and noninvasive arterial blood pressure were applied. General anesthesia was induced with midazolam 0.05 mg/kg, etomidate 0.1 mg/kg, propofol 2 mg/kg, sufentanil 0.5 *μ*g/kg, and cisatracurium 2 mg/kg. Propofol 4–12 mg/kg/h and remifentanil 5–40 *μ*g/kg/h were infused through micro pumps to maintain anesthesia. These drugs' dose was adjusted according to the change of hemodynamics. The neuromuscular blockade was maintained by intermittent injection of cisatracurium as required.

After the surgery, all the patients received patient-controlled intravenous analgesia (PCIA) when extubated and then were transferred to the postanesthesia recovery room. No other analgesics were given during the perioperative period. A physician who was blinded to the group assignment assessed spontaneous postsurgical pain intensity at rest using VAS at 1, 2, 4, 6, 12, 24, and 48 hours after the operation.

### 2.4. Assessment of Emergence Agitation Score and the Ramsay Calm Score

Another physician who was blinded to the group assignment assessed emergence agitation score and the Ramsay calm score at 0, 5, 10, 30, and 60 min after extubation. The emergence agitation score is assessed as follows: 0 points is cooperation, 1 point is physical restlessness when stimulated, 2 points is slight physical restlessness without stimulation, and 3 points is fierce struggling and difficult to control. The Ramsay calm score is assessed as 1 point is anxiety, 2 points is cooperation, 3 points is drowsiness but obedience to instructions, 4 points is asleep but can wake up easily, 5 points is asleep but can react to strong stimulation, and 6 points is deeply asleep and cannot be woken up (2–4 points display sedation well; 5-6 points display over sedation).

### 2.5. Determination of the Levels of Systemic Proinflammatory Cytokines

Blood (2 mL) was collected from the central vein before induction of anesthesia, at the end of operation, and 24 h and 48 h after the surgery. Plasma was separated by centrifugation at 3000 rpm for 10 min and stored at −70°C for further analysis. Plasma inflammatory cytokines tumor necrosis factor- (TNF-) a and IL-6 levels were measured, using the commercially available human ELISA kit (Jiancheng Co., Nanjing, China) as described [19]. All steps involved were operated according to the manufacturers' instructions.

### 2.6. Statistical Analysis

Statistical analysis was performed using SPASS 16.0 software. Values were expressed as mean ± SD, with one-way ANOVA and *q* test used among the groups and chi-square test used on counting data. *P* < 0.05 was considered statistically significant.

## 3. Results

Ninety patients were involved in the present study. One patient from group A did not have his scheduled surgery; two patients from group C were excluded because of postoperative transfer to another hospital. As shown in Table 1, there were no significant differences among the three groups in terms of gender, weight, height, operation time, and blood loss. In addition, heart rate, arterial blood pressures, pulse oximetry, and fluid administration were not significantly different among the three groups (*P* > 0.05). Postoperative tramadol consumption in group A was significantly lower than that in group C but significantly higher than that in group B (*P* < 0.05).

**Figure 1:**
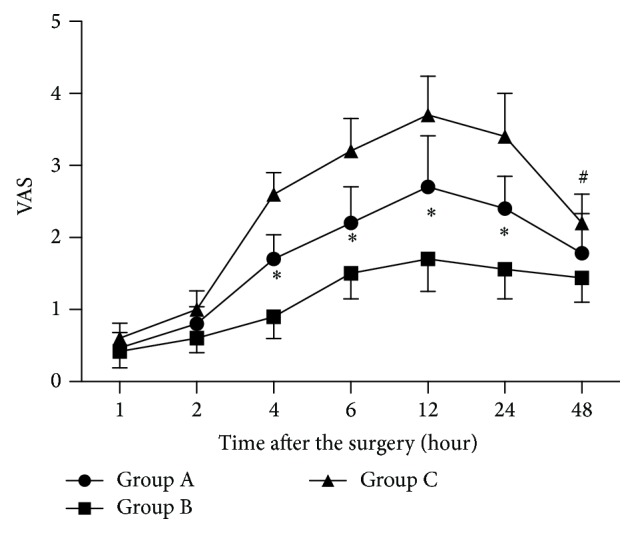
Visual analog pain scale: patients rated their levels of pain on the 0–10 cm VAS (0 cm = no pain to 10 cm = the worst possible pain). ^*^ *P* < 0.05 versus other groups, ^#^ *P* < 0.05 group C versus group B.

VAS data are presented in Figure 1. There were no significant differences among groups A, B, and C at 1 and 2 h after surgery (*P* > 0.05). VAS in groups A and B were significantly lower than that in group C at 4, 6, 12, and 24 h after surgery (*P* < 0.05), and the VAS in group B is also significantly lower than that of group A at 4, 6, 12, and 24 h after surgery (*P* < 0.05). Further, the VAS in group C is significantly higher than that in group B at 48 h after the surgery (*P* < 0.05), but without significant increase as compared with that of group A (*P* > 0.05).

To further investigate the efficacy of flurbiprofen axetil, we assessed the emergence agitation score and Ramsay calm score. As shown in Tables 2 and 3, the emergency agitation score and Ramsay score at 0, 5 and 10 min after extubation in groups A and B were significantly lower than that in group C. There was no significant difference between groups A and B at 0, 5, and 10 min after extubation, and no significant difference among the three groups at 30 minutes and 60 minutes after extubation was observed.

Then, we investigate the anti-inflammatory effects of flurbiprofen axetil in patients undergoing tangential excision surgery. As shown in Tables 4 and 5, operation significantly increased the plasma levels of TNF-*α* and IL-6, which was significantly decreased by preoperative administration of flurbiprofen axetil at 24 and 48 h after the operation. Postoperative administration of flurbiprofen axetil further significantly reduced the plasma levels of TNF-*α* and IL-6 (*P* < 0.05, group A versus group B) at 24 and 48 h after the operation.

## 4. Discussion

In the present study, we demonstrated that flurbiprofen axetil improves analgesic effects of sufentanil. This is well supported by our results showing that preoperative administration of flurbiprofen axetil decreased the VAS at 4, 6, 12, and 24 h after surgery, and the VAS was further decreased by postoperative administration of flurbiprofen axetil in PCIA pump, which is associated with lower postoperative tramadol consumption. Furthermore, flurbiprofen axetil attenuated postoperative emergence agitation and reduced the systemic levels of proinflammatory cytokines. To the best of our knowledge, this is the first study to investigate the efficiency of flurbiprofen axetil on postoperative pain and emergence agitation.

Emergence agitation is a postanesthetic complication that interferes with a patient's recovery and presents a challenge in terms of assessment and management. It mainly occurs within 15 min after extubation [20]. Although the pathophysiology mechanisms of this complication are very complicated, much evidence indicates the involvement of pain stimulus [21–23]. Thus, all kinds of analgesics [24, 25] have been used in experimental and clinical studies to manage the occurrence of emergence agitation, among which were preemptive analgesics such as dexmedetomidine, clonidine, opioids, ketorolac, and lornoxicam. Our present study further confirmed that modulation of postoperative pain relief attenuates emergence agitation. This is evidenced by flurbiprofen axetil mediated reduction in emergence agitation score and Ramsay calm score at 0, 5, and 10 min after extubation.

Preemptive analgesia, a new concept to enhance the postoperative analgesia, is to give a first dose of analgesics before pain stimulation. Nevertheless, some clinical studies have conflicting results regarding the efficacy of preemptive analgesia [26]. However, Mihara and colleagues [27] evaluated the efficacy of flurbiprofen axetil for postoperative pain management after laparoscopic colectomy, and the conclusion was satisfactory. Similar to other studies [15, 28], our present study suggested preoperative administration of flurbiprofen axetil had preemptive analgesia effects, which was supported by the results that flurbiprofen axetil decreased the VAS at 4, 6, 12, and 24 h after surgery and postoperative tramadol consumption. Furthermore, postoperative administration of flurbiprofen axetil in PCIA pump enhanced the analgesic effects of sufentanil.

The burn patient's pain, infection, and other factors weaken the body's immune status and predispose the patients to sepsis and multiple organ failure [5, 6]. The proinflammatory cytokines after burn injury play an important role in the development of these complications. TNF-*α* is believed to be the initiating cytokine that induces a cascade of secondary cytokines and humoral factors that can lead to local and systemic sequelae [29]. Moreover, TNF-*α* is a potent mediator of the shock-like state associated with thermal injury and sepsis [30]. In the present study, tangential excision surgery significantly increased the plasma levels of TNF-*α* and IL-6, which was decreased by preoperative administration of flurbiprofen axetil, and further reduced by postoperative administration of flurbiprofen axetil at 24 and 48 h after the operation.

The NSAIDs are associated with many adverse effects, including reducing platelet aggregation, renal and gastrointestinal mucosal injury. In our present study, there were no adverse effects found in any of the involved patients, which might be due to the relatively short period of flurbiprofen axetil administration in the current study. However, there are still several limitations in the current study, such as psychosocial characteristics, educational background, and preoperative pathology of the patients that were not controlled, and it is also not well known in our study whether pain relief attenuates the postoperative systemic inflammatory response, or the decrease of inflammatory response reduces postoperative pain.

## 5. Conclusion

In summary, the results of current study demonstrate that flurbiprofen axetil can enhance analgesic effects of sufentanil and attenuate emergence agitation, which was associated with decrease of systemic proinflammatory cytokines release in patients undergoing tangential excision surgery. Our data suggest that intravenous flurbiprofen axetil may be useful approaches to reduce postoperative pain and emergence agitation in burn patients.

## Figures and Tables

**Table 1:** General data of the three groups (mean ± SD).

Items	Group A (*n* = 29)	Group B (*n* = 30)	Group C (*n* = 28)
Gender (M/F)	17/12	19/11	16/12
Age (years)	41.2 ± 7.1	39.2 ± 8.3	40.6 ± 6.9
Weight (kg)	62.3 ± 11.8	66.2 ± 13.1	65.8 ± 10.6
Height (cm)	166 ± 7	167 ± 5	165 ± 8
Operation time (min)	112.8 ± 24.5	118.3 ± 27.8	116.7 ± 21.2
Blood loss (mL)	386 ± 107	413 ± 125	409 ± 114
Postoperative sufentanil (analgesic) dose (*μ*g)	125.6 ± 23.5	132.4 ± 26.3	130.6 ± 21.2
Postoperative tramadol requirement dose (mg)	147.7 ± 30.3^*^	68.5 ± 20.4^∗∗#^	230.5 ± 39.6

^*^ *P* < 0.05, ^**^ *P *< 0.05 versus group C, ^#^ *P* < 0.05 versus group A.

**Table 2:** Emergence agitation score among the three groups (mean ± SD).

Groups	Time after extubation (min)				
	0	5	10	30	60
A (*n* = 29)	1.33 ± 0.41	1.01 ± 0.52	0.87 ± 0.16	0.58 ± 0.62	0.00 ± 0.00
B (*n* = 30)	1.31 ± 0.32	0.97 ± 0.47	0.84 ± 0.09	0.55 ± 0.64	0.00 ± 0.00
C (*n* = 28)	2.82 ± 0.21^*^	2.71 ± 0.45^*^	2.07 ± 0.51^*^	0.60 ± 0.28	0.00 ± 0.00

^*^ *P* < 0.05 versus groups A and B.

**Table 3:** Ramsay calm score among the three groups (mean ± SD).

Groups	Time after extubation (min)				
	0	5	10	30	60
A (*n* = 29)	2.24 ± 0.34	2.38 ± 0.43	2.02 ± 0.65	2.01 ± 0.21	2.00 ± 0.00
B (*n* = 30)	2.31 ± 0.72	2.21 ± 0.44	2.77 ± 0.38	1.93 ± 0.22	2.00 ± 0.00
C (*n* = 28)	3.47 ± 0.26^*^	3.36 ± 0.36^*^	3.85 ± 0.22^*^	1.92 ± 0.34	2.00 ± 0.00

^*^ *P* < 0.05 versus groups A and B.

**Table 4:** Plasma level of TNF-*α* (pg/mL) among the three groups (mean ± SD).

Groups	Before induction of anesthesia	Time after operation (h)		
		0	24	48
A (*n* = 29)	547.56 ± 36.73	956.42 ± 32.19	780.62 ± 35.71^∗$^	738.37 ± 27.85^$^
B (*n* = 30)	570.91 ± 31.53	961.45 ± 34.17	658.87 ± 28.35^∗∗#$^	612.64 ± 21.31^∗#$^
C (*n* = 28)	554.64 ± 37.36^$^	948.35 ± 39.78	890.68 ± 31.72	806.10 ± 32.62

^*^ *P* < 0.05, ^**^ *P* < 0.05 versus group C; ^#^ *P* < 0.05 versus group A.

^$^ *P* < 0.05 versus all the other time points in the patients of the same group.

**Table 5:** Plasma level of IL-6 (pg/mL) among the three groups (mean ± SD).

Groups	Before induction of anesthesia	Time after operation (h)		
		0	24	48
A (*n* = 29)	220.33 ± 13.16	359.45 ± 17.36	283.38 ± 12.32^∗$^	263.31 ± 19.20^$^
B (*n* = 30)	218.68 ± 15.42	351.38 ± 16.38	236.67 ± 13.49^∗∗#$^	229.38 ± 15.49^∗#$^
C (*n* = 28)	225.73 ± 14.18^$^	349.95 ± 17.81	327.28 ± 20.18	289.38 ± 17.73

^*^ *P* < 0.05, ^**^ *P* < 0.05 versus group C; ^#^ *P* < 0.05 versus group A.

^$^ *P* < 0.05 versus all the other time points in the patients of the same group.
